# Inhibitory Effects of AF-343, a Mixture of *Cassia tora* L., *Ulmus pumila* L., and *Taraxacum officinale*, on Compound 48/80-Mediated Allergic Responses in RBL-2H3 Cells

**DOI:** 10.3390/molecules25102434

**Published:** 2020-05-22

**Authors:** Eun Kyeong Lee, Jeongah Song, Youjin Seo, Eun Mi Koh, Seon-Hee Kim, Kyung Jin Jung

**Affiliations:** 1 Bioanalytical and Immunoanalytical Research Group, Korea Institute of Toxicology, 141 Gajungro, Yuseong-gu, Daejeon 34114, Korea; eunkyeong.lee@kitox.re.kr (E.K.L.); emkoh@kitox.re.kr (E.M.K.); 2 Animal Model Research Group, Korea Institute of Toxicology, Jeongeup, Jeonbuk 580-185, Korea; jasong@kitox.re.kr; 3 Jeonbuk Analytical Research Group, Korea Institute of Toxicology, Jeongeup, Jeonbuk 580-185, Korea; youjin.seo@kitox.re.kr; 4 Sungkyun Biotech Co., Ltd., SungKyunKwan University, #2066, Seobu-ro, Jangan-gu, Suwon-si, Gyeonggi-do 61256, Korea; seonhee31@gmail.com

**Keywords:** AF-343, *Cassia tora* L., *Ulmus pumila* L., *Taraxacum officinale*, antiallergic responses, compound 48/80, RBL-2H3 cells

## Abstract

The purpose of this study was to determine the antiallergic effects of AF-343, a mixture of natural plant extracts from *Cassia tora* L., *Ulmus pumila* L., and *Taraxacum officinale*, on rat basophilic leukemia (RBL-2H3) cells. The inhibitory effects on cell degranulation, proinflammatory cytokine secretion, and reactive oxygen species (ROS) production were studied in compound 48/80-treated RBL-2H3 cells. The bioactive compounds in AF-343 were also identified by HPLC–UV. AF-343 was found to effectively suppress compound 48/80-induced β-hexosaminidase release, and interleukin (IL)-4 and tumor necrosis factor-α (TNF-α) production in RBL-2H3 cells. In addition, AF-343 exhibited DPPH free radical scavenging effects in vitro (half-maximal inhibitory concentration (IC_50_) = 105 μg/mL) and potently inhibited compound 48/80-induced cellular ROS generation in a 2′,7′-dichlorofluorescein diacetate (DCFH-DA) assay. Specifically, treatment with AF-343 exerted stronger antioxidant effects in vitro and antiallergic effects in cells than treatment with three single natural plant extracts. Furthermore, AF-343 was observed to contain bioactive compounds, including catechin, aurantio-obtusin, and chicoric acid, which have been reported to elicit antiallergic responses. This study reveals that AF-343 attenuates allergic responses via suppression of β-hexosaminidase release, IL-4 and TNF-α secretion, and ROS generation, perhaps through mechanisms related to catechin, aurantio-obtusin, and chicoric acid. The results indicate that AF-343 can be considered a treatment for various allergic diseases.

## 1. Introduction

Allergic reactions are exaggerated or abnormal responses caused by hypersensitivity of the immune system to foreign substances known as allergens, which can include certain foods, drugs, insect products, and plant pollens [1]. Persistent or repetitive exposure to allergens can result in allergic diseases, including food allergies, atopic dermatitis, allergic asthma, and anaphylaxis, which now afflict approximately 30–35% of the population [2].

Allergic diseases are caused by inappropriate activation of T helper 2 (Th2) cells [3]. Th2 cell-mediated recognition of allergens carried by antigen-presenting cells triggers the release of cytokines, including interleukin (IL)-3, IL-4, IL-5, and IL-13, which leads to the secretion of allergen-specific molecules known as immunoglobulin E (IgE) antibodies. These antibodies attach to receptors on mast cells in tissue and on basophils circulating in blood and then activate the cells to release β-hexosaminidase and histamine in a process called degranulation; the antibodies also induce secretion of a number of inflammatory mediators, including tumor necrosis factor-α (TNF-α), IL-4, IL-6, and IL-8 [4]. These secreted cytokines promote the expression and activity of adhesion molecules in vascular endothelial cells, thereby promoting the movement of basophils, eosinophils, and other cells, leading to tissue damage and apoptosis [5]. Mast cells are known to degranulate in response to synthetic compounds, such as compound 48/80, melittin, polyamine, codeine, and the calcium ionophore A23187, and these compounds have been used as convenient reagents for direct studies of the mechanisms of in vitro allergy responses [6,7,8]. Thus, this study investigated the antiallergic effects of a natural plant extract, AF-343, on compound 48/80-challenged rat basophilic leukemia (RBL-2H3) cells. 

RBL-2H3 cells are a rat basophilic leukemia cell line isolated from Wistar rat basophilic cells. However, RBL-2H3 cells are known to present some typical characteristics of both mast cells and basophils [9]. RBL-2H3 cells have been widely used by many groups because they have the functional characteristics of mast cells involving IgE–Fc epsilon RI (FcεRI) interactions, degranulation, and inflammatory cytokine production [10,11]. Therefore, RBL-2H3 cells may be useful as models for research on the effects of unknown compounds on β-hexosaminidase release and cytokine secretion.

New drugs based on natural products have been continuously developed because they are less toxic and more stable than chemical drugs [12]. A variety of studies based on natural products have been conducted in major therapeutic fields, such as the immunosuppression, anti-infection, and metabolic disease fields [13]. Representative natural product drugs include aspirin; Tamiflu, a treatment for influenza; and Prograf, an immunosuppressant [14].

AF-343 is a mixture of natural plant extracts from *Ulmus pumila* L., *Cassia tora* L., and *Taraxacum officinale*. AF-343 is known to exhibit anti-inflammatory and skin hydration effects in HS68 cells and in NC/Nga mouse models of atopic dermatitis [15]. Despite the existence of reported data showing the anti-inflammatory activity of AF-343, the compound has not been fully examined for its antiallergic effects in RBL-2H3 cells.

Therefore, the purpose of this study was to elucidate the antiallergic effects of AF-343 in RBL-2H3 cells. We assessed β-hexosaminidase release, proinflammatory cytokine (including IL-4 and TNF-α) secretion, and reactive oxygen species (ROS) generation in the presence of AF-343. Furthermore, we analyzed the bioactive components of AF-343, such as chicoric acid, aurantio-obtusin, and catechin. Our findings provide new insights into the mechanisms of the antiallergic actions of AF-343 and support the application of AF-343 in allergic disease treatment.

## 2. Results and Discussion

### 2.1. Cytotoxicity of AF-343 toward RBL-2H3 Cells

The activity of AF-343 was compared with that of the individual components of AF-343 (*Ulmus pumila* L., *Cassia tora* L., and *Taraxacum officinale*) in all experiments. To investigate the cytotoxic effects of AF-343 and the extracts of *Ulmus pumila* L., *Cassia tora* L., and *Taraxacum officinale*, RBL-2H3 cells were treated with various concentrations (5, 10, 50, 100, 250, and 500 μg/mL) of the substances for 24 h and then subjected to a Cell Counting Kit (CCK)-8 assay.

**Figure 1:**
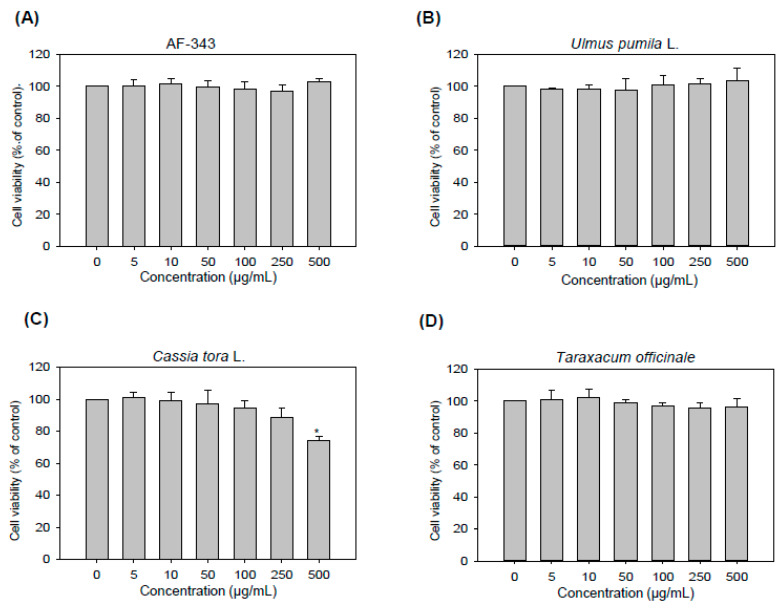
Cytotoxicity of AF-343 and the individual extract components toward rat basophilic leukemia (RBL-2H3) cells. (**A**–**D**) RBL-2H3 cells were treated with various concentrations (5, 10, 50, 100, 250, and 500 μg/mL) of AF-343 and extracts of *Ulmus pumila* L., *Cassia tora* L. and *Taraxacum officinale* for 48 h. Cell viability was measured using a Cell Counting Kit (CCK)-8 assay. The results are expressed as the mean ± SD from three independent experiments. * *p* < 0.05 vs. the compound 48/80 only group.

The results showed that AF-343 did not exert cytotoxicity at any concentration (Figure 1A). Additionally, the *Ulmus pumila* L. and *Taraxacum officinale* extracts did not exert cytotoxicity at any concentrations (Figure 1B,D). However, the *Cassia tora* L. extract inhibited RBL-2H3 cell growth by 20% at the 500 μg/mL concentration (Figure 1C).

### 2.2. Inhibitory Effects of AF-343 on Compound 48/80-Induced Degranulation in RBL-2H3 Cells 

It has been reported that allergic diseases involve type I hypersensitivity reactions mediated by the activity of mast cells that result in degranulation and inflammatory mediator release [1]. Degranulation is an essential symptom of mast cell-mediated allergic reactions and is thus considered a key target for antiallergic compounds [16]. Here, we explored the possible mechanism by which AF-343 inhibits compound 48/80-induced allergic responses in RBL-2H3 cells. 

**Figure 2:**
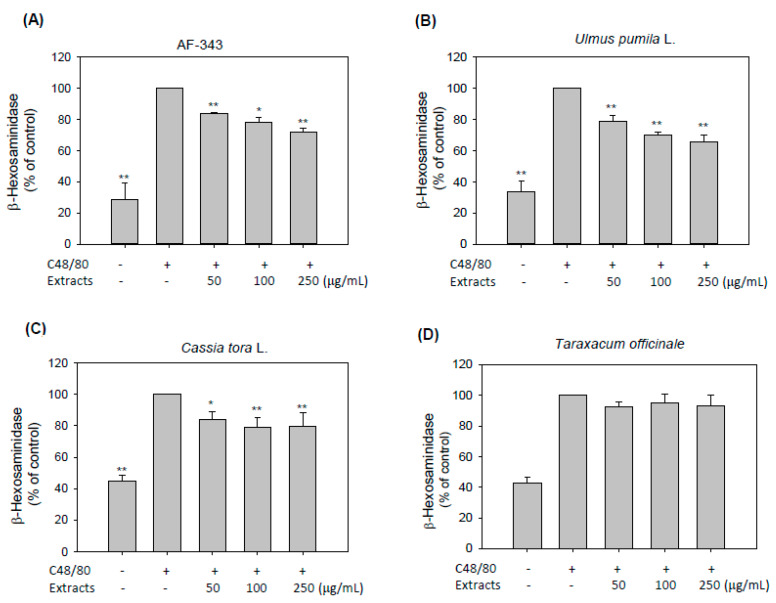
Inhibitory effects of AF-343 and extracts of *Ulmus pumila* L., *Cassia tora* L., and *Taraxacum officinale* on β-hexosaminidase release in RBL-2H3 cells. (**A**–**D**) RBL-2H3 cells were pretreated with the test samples for 1 h following stimulation with compound 48/80 for 1 h. β-hexosaminidase release was detected in the cell culture supernatants. The results are expressed as the mean ± SD from three independent experiments. * *p* < 0.05 and ** *p* < 0.01 vs. the compound 48/80 only group. C48/80, compound 48/80.

First, we measured the release of β-hexosaminidase as a degranulation indicator. Our study showed that AF-343 significantly inhibited degranulation in compound 48/80-challenged RBL-2H3 cells in a dose-dependent manner (Figure 2A). Additionally, the *Ulmus pumila* L. and *Cassia tora* L. extracts significantly decreased β-hexosaminidase release at all doses (Figure 2B,C). However, the *Taraxacum officinale* extract did not decrease β-hexosaminidase release at any dose (Figure 2D). These data indicate that the inhibitory effects of AF-343 on mast cell degranulation are due to the *Ulmus pumila* L. and *Cassia tora* L. extracts.

### 2.3. Ameliorative Effects of AF-343 on Compound 48/80-Mediated Cytokine Production in RBL-2H3 Cells 

Next, we investigated the suppressive effect of AF-343 on cytokine production by compound 48/80-challenged RBL-2H3 cells. Activated mast cells secrete β-hexosaminidase as well as multiple cytokines, including TNF-α, IL-4, IL-5, IL-6, IL-10, and IL-13 [17]. These cytokines promote local and systemic inflammation by enhancing the recruitment and infiltration of leukocytes and lymphocytes into sites of inflammation, further accelerating allergic disease progression [18]. 

**Figure 3:**
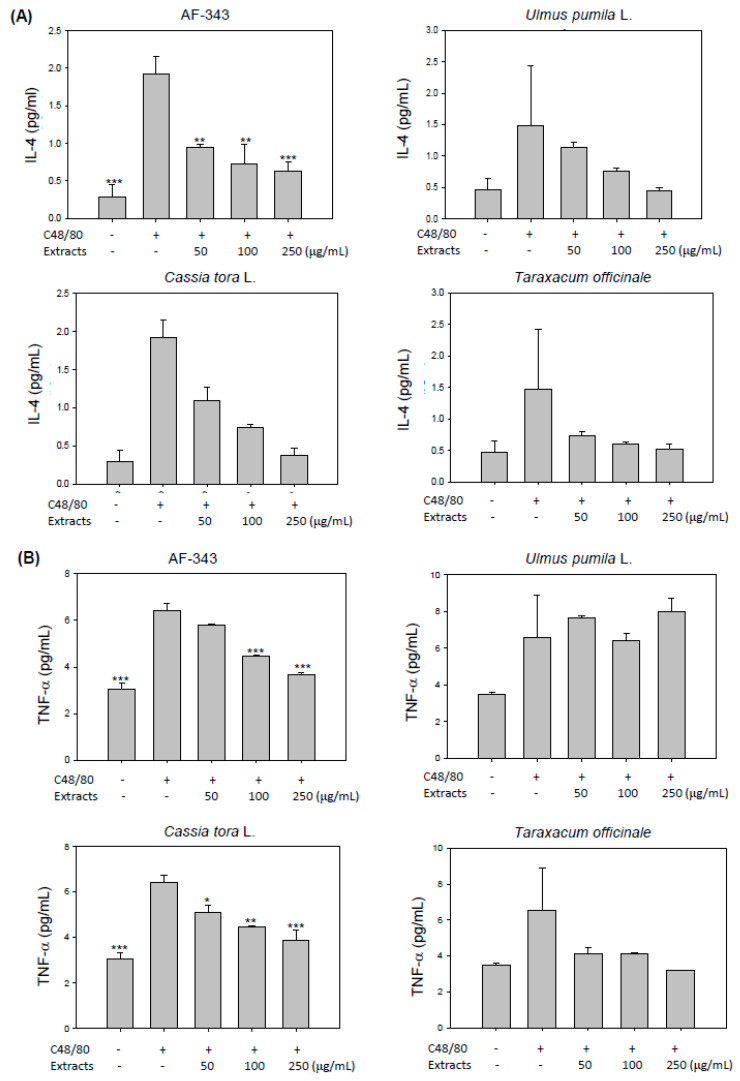
Ameliorative effects of AF-343 and extracts of *Ulmus pumila* L., *Cassia tora* L., and *Taraxacum officinale* on compound 48/80-induced cytokine release in RBL-2H3 cells. (**A** and **B**) Supernatants were taken after 24 h of incubation for determination of cytokine concentrations using a V-PLEX Proinflammatory Panel 2 (Rat) Kit. The results are expressed as the means ± SD from three independent experiments. * *p* < 0.05, ** *p* < 0.01 and *** *p* < 0.001 vs. the compound 48/80 only group. C48/80, compound 48/80.

To determine whether AF-343 modulates compound 48/80-mediated cytokine production, we examined the release of nine kinds of cytokines (IFN-γ, IL-1β, IL-4, IL-5, IL-6, KC/GRO, IL-10, IL-13, and TNF-α) using a V-PLEX Proinflammatory Panel 2 Kit. The results showed that the production of IL-4 and TNF-α in compound 48/80-treated RBL-2H3 cells was higher than that in untreated control RBL-2H3 cells (Figure 3A,B). However, the production of IFN-γ, IL-5, IL-6, IL-10, and IL-13 was unchanged by compound 48/80 treatment (unpublished data), and secretion of IL-1β and KC/GRO was not detected after compound 48/80 treatment. On the other hand, AF-343 significantly inhibited the production of IL-4 and TNF-α in a dose-dependent manner. Additionally, the *Cassia tora* L. and *Taraxacum officinale* extracts inhibited the secretion of IL-4 and TNF-α, but the *Ulmus pumila* L. extract decreased the secretion only of IL-4 (Figure 3A,B). These results suggest that IL-4 and TNF-α are important cytokines for mediating allergic responses in compound 48/80-treated RBL-2H3 cells and that AF-343 may help ameliorate allergic responses through inhibition of the release of these cytokines.

Mast cells possess the secretory granules containing histamine, serotonin, and other inflammatory mediators, such as IL-6, IL-4, TNF-α, and MIP-1α. Within seconds of activation, mast cells secrete these mediators via massive exocytosis, accompanied by both immediate- and late-phase inflammatory responses. Mast cells are considered the only cells capable of storing preformed cytokine TNF-α in cytoplasmic granules and rapidly releasing it upon activation. However, the release process of TNF-α from cytoplasmic granules of mast cells is known to be modulated by a mechanism distinct from that of degranulation [19,20]. Granule exocytosis in mast cells is controlled by membrane–membrane fusion proteins termed VAMP (vesicle-associated membrane protein). Among several VAMP proteins, VAMP-8 is a key regulator of β-hexosaminidase and histamine release from mast cells, but VAMP-8 does not affect cytokine release, indicating that cytokine secretion uses distinct trafficking pathways independent of VAMP-8 [19]. Therefore, our findings from Figure 2 and Figure 3 demonstrate that AF-343 inhibits secretion of β-hexosaminidase and TNF-α in compound 48/80-treated mast cells, but exact regulatory mechanism of AF-343 on mediators secretion in cytoplasmic granules requires further investigation. 

### 2.4. Antioxidant Activity of AF-343 In Vitro and in a Cell Culture System

ROS are promoters of allergic sensitization, and the incidence of allergic diseases such as asthma, rhinitis, and atopic dermatitis are associated with oxidative stress [21]. ROS in sensitized cells act as intracellular second messengers in allergic responses, inducing inflammatory mediator production, and related signal transduction [22]. To examine the antioxidative ability of AF-343, we evaluated the ROS scavenging activity of AF-343 using 2,2-di(4-tert-octylphenyl)-1-picrylhydrazyl (DPPH) and cellular 2′,7′-dichlorofluorescein diacetate (DCFH-DA) assays. DPPH is a stable free radical, which is widely used to measure the free radical scavenging activity of various extracts [23]. DPPH is a dark-purple organic nitrogen radical with a maximum absorbance at 517 nm wavelength that is reduced in the presence of antioxidants [24]. 

**Figure 4:**
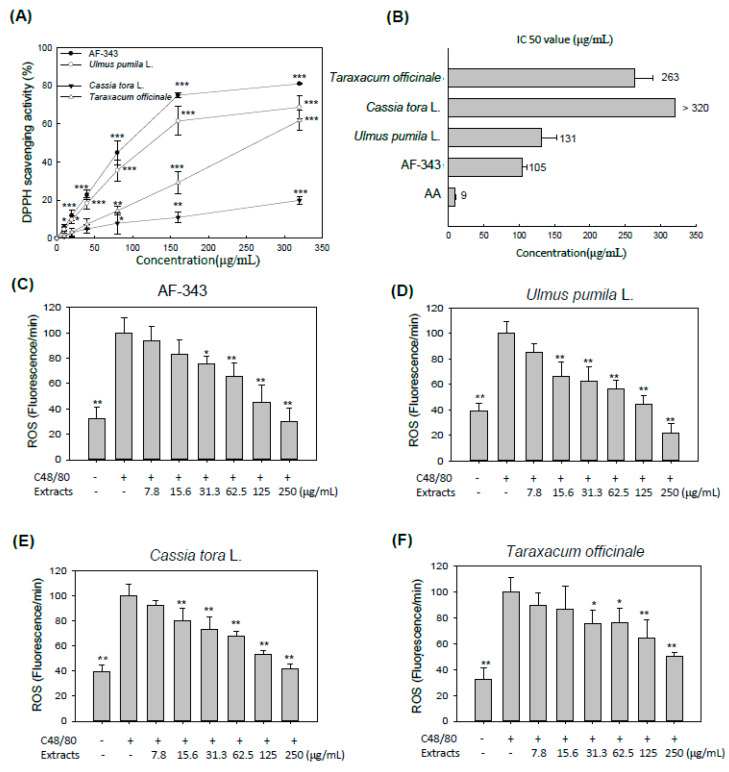
Antioxidative effects of AF-343 and extracts of *Ulmus pumila* L., *Cassia tora* L., and *Taraxacum officinale* on RBL-2H3 cells. (**A**) DPPH scavenging activity and (**B**) half-maximal inhibitory concentration (IC_50_) values of AF-343, and extracts of *Ulmus pumila* L., *Cassia tora* L., and *Taraxacum officinale* at various concentrations (10, 20, 40, 80, 160, and 320 μg/mL). The results are expressed as the means ± SD from three independent experiments. AA, ascorbic acid. (**C**–**F**) RBL-2H3 cells were preincubated with various concentrations (7.8, 15.6, 31.3, 62.5, 125, and 250 μg/mL) of AF-343 or *Ulmus pumila* L., *Cassia tora* L., or *Taraxacum officinale* extract for 1 h. The cells were treated with 500 μg/mL compound 48/80 for an additional 30 min. Changes in ROS levels were detected after the cells were treated with 2′,7′-dichlorofluorescein diacetate (DCFH-DA) (25 μM), an oxidant sensing probe. The results are expressed as the mean ± SD of three independent experiments. * *p* < 0.05 and ** *p* < 0.01 vs. the compound 48/80 only group; C48/80, compound 48/80.

The DPPH free radical scavenging activity and half-maximal inhibitory concentration (IC_50_) values for various concentrations (10, 20, 40, 80, 160, and 320 μg/mL) of AF-343 and the individual extract components of AF-343 (*Ulmus pumila* L., *Cassia tora* L., and *Taraxacum officinale*) are shown in Figure 4A,B, respectively. AF-343 showed more potent antioxidant activity than the three individual extracts, and had an IC_50_ value of 105 μg/mL. The *Ulmus pumila* L. and *Taraxacum officinale* extracts also showed dose-dependent DPPH free radical scavenging activity and exhibited IC_50_ values of 131 and 263 μg/mL, respectively. However, *Cassia tora* L. showed low DPPH free radical scavenging activity at concentrations from 10 to 320 μg/mL (IC_50_ > 320 μg/mL). Ascorbic acid was used as a positive control.

The DCFH-DA assay is a fluorometric assay in which a DCFH-DA probe is used to measure the degree of antioxidant capacity for scavenging of oxidative stress-induced free radicals in cells [25]. Figure 4C–F shows the cellular antioxidant activity of AF-343 and the individual extract components of AF-343. The results showed that AF-343 significantly suppressed compound 48/80-induced ROS generation in a dose-dependent manner (Figure 4C). In addition, all three individual extracts of AF-343 showed a significant antioxidant ability by decreasing compound 48/80-induced ROS generation (Figure 4D–F). The *Cassia tora* L. extract showed lower antioxidant activity than the other extracts in the DPPH scavenging assay, but it showed an antioxidant ability similar to that of AF-343 and the other two individual extract components of AF-343 in the intracellular assay. This difference is due to the fact that antioxidants act through a number of mechanisms in vivo and in cells but act through only simple chemical reactions in vitro [26].

The inhibitory effect of *Ulmus pumila* L. and *Cassia tora* L. extracts on mast cell degranulation is similar to that of AF-343. However, *Ulmus pumila* L. extracts treatment did not prevent TNF-α production, and *Cassia tora* L. extracts have cytotoxicity at the 500 μg/mL concentration. In addition, three individual extracts have weaker antioxidant ability in vitro than AF-343. Therefore, AF-343 is a more effective treatment for antiallergic response than three single natural plant extracts.

We found that the AF-343 used in the present study contained catechin, aurantio-obtusin, and chicoric acid, which are well-known bioactive compounds of *Ulmus pumila* L., *Cassia tora* L., and *Taraxacum officinale* extracts, respectively (Table 1). The chromatograms of catechin, aurantio-obtusin, and chicoric acid identified in the three individual extracts of AF-343 are shown in Supplementary Figure S1–S3. Catechin is isolated from the root of *Ulmus pumila* L. and has potent hypocholesterolemic, antihypertensive, and antioxidant functions [27,28,29]. Aurantio-obtusin is a predominant anthraquinone compound in dried seeds of *Cassia tora* L. and appears to have various biological properties, including antiallergic, anti-inflammatory, and hypolipidemic properties [30,31,32]. Chicoric acid isolated from *Taraxacum officinale* is a phenolic compound that has been shown to have anti-inflammatory, antioxidative, and antiallergic activity [33,34,35]. Thus, the most powerful antiallergic activity of AF-343 is thought to originate from catechin, aurantio-obtusin, and chicoric acid, although some of the compounds of AF-343 have yet to be identified.

## 3. Conclusions

This study demonstrated that AF-343, a mixture of natural plant extracts, prevented activation of compound 48/80-treated mast cells by inhibiting β-hexosaminidase release, IL-4 and TNF-α production, and ROS generation. In particular, the antiallergic effects of AF-343 were greater than those of *Ulmus pumila* L., *Cassia tora* L., and *Taraxacum officinale*. The antiallergic activity of AF-343 may be related to the effectiveness of its bioactive compounds, including chicoric acid, aurantio-obtusin, and catechin, which have antiallergic and anti-inflammatory properties. The results provide insight into the mechanism responsible for the antiallergic activity of AF-343 and suggest that AF-343 is a natural product candidate for the preventive treatment of mast cell-induced allergic diseases.

## 4. Materials and Methods

### 4.1. Plant Materials and Chemicals

AF-343 was supplied by the manufacturer Sungkyun Biotech Co., Ltd. (Suwon-si, Rep. of Korea). The AF-343 was produced as a mixture of 3 natural plant extracts, including extracts from *Ulmus pumila* L., *Cassia tora* L., and *Taraxacum officinale,* via hot-water extraction, concentration, and powdering.

All chemicals and reagents, including compound 48/80, 4-nitrophenyl-N-acetyl-β-D-glucosaminide, chicoric acid, catechin, aurantio-obtusin, and DPPH, were obtained from Sigma-Aldrich (St. Louis, MO), except where otherwise noted. A CCK-8 assay was purchased from Dojindo Laboratories (Kumamoto, Japan). DCFH-DA was purchased from Molecular Probes (Eugene, OR). All other materials used were of the highest available grade.

### 4.2. Cell Line and Culture Conditions 

Rat basophilic leukemia cells of the RBL-2H3 cell line were acquired from the American Type Culture Collection (ATCC, Manassas, VA). The RBL-2H3 cells were kept in Eagle’s Minimum Essential Medium (EMEM) containing 10% (v/v) heat-inactivated fetal bovine serum (FBS), penicillin (50 U/mL), and streptomycin (50 μg/mL). The EMEM was purchased from the ATCC (Gaithersburg, MD), and the supplements were obtained from Gibco Invitrogen (Grand Island, NY). The cells were cultured in plastic tissue culture flasks and passaged three times weekly.

### 4.3. Cell Viability Assay

For the CCK-8 assay, RBL-2H3 cells were seeded into flat-bottomed 96-well plates and allowed to stabilize for 24 h. The cells were then treated with various concentrations of AF-343 and individual of extracts of *Ulmus pumila* L., *Cassia tora* L., and *Taraxacum officinale* for 48 h. CCK-8 reagent was then added to each well, and the cells were further incubated for 2 h. A colorimetric assay was performed using a SpectraMax M3 microplate reader (Molecular Devices, Sunnyvale, CA) at 450 nm. 

### 4.4. Cell Degranulation

To assess mast cell degranulation, β-hexosaminidase release was evaluated according to a method published by Dearman et al. (2005) [36]. Briefly, RBL-2H3 cells were seeded in 24-well plates (2.5 × 10^5^ cells/well). After incubation for 24 h, the cells were washed twice with Siraganian buffer (119 mM NaCl, 5 mM KCl, 5.6 mM glucose, 0.4 m8M MgCl_2_, 1 mM CaCl_2_, 25 mM PIPES, and 0.1% bovine serum albumin, pH 7.2) and then incubated in 200 μL of Siraganian buffer for 10 min at 37 °C. Next, 100 μL of test sample solution was added to each well, and the plates were incubated for 1 h at 37 ℃. To stimulate cell degranulation, 100 μL of compound 48/80 (500 μg/mL) was added, and the plates were incubated for an additional 1 h at 37 °C. The supernatant (20 μL) was transferred into a 96-well plate, and 80 μL of 1 mM p-nitrophenyl-N-acetyl-β-D-glucosaminide was added. After incubation for 1 h, the enzyme reaction was stopped by adding 200 μL of 0.1 M sodium carbonate buffer (pH 10.0), and the absorbance was determined at 405 nm using a SpectraMax M3 microplate reader (Molecular Devices). 

### 4.5. Determination of Proinflammatory Cytokines Levels 

RBL-2H3 cells were pretreated with various concentrations of AF-343; *Ulmus pumila* L., *Cassia tora* L., or *Taraxacum officinale* extract; or vehicle at 37 °C for 1 h and incubated with compound 48/80 (10 μg/mL) for 24 h. A total of 100 µL of supernatant was used for a highly sensitive multiplex enzyme-linked immunosorbent assay (ELISA) with a Merck Sharp and Dohme Co. (MSD) V-PLEX Proinflammatory Panel 2 (Rat) Kit, which can simultaneously measure IFN-γ, IL-1β, IL-4, IL-5, IL-6, KC/GRO, IL-10, IL-13, and TNF-α using an electrochemiluminescence detection method (Meso Scale Diagnostics, LLC., Rockville, MD). The samples and standards were briefly added to 10-spot MULTI-SPOT plates. The lowest limit of detection (LLOD) was calculated according to the manufacturer’s protocol, and the mean value for the plate was used for further calculation of the sample concentrations. Any value below the LLOD for the cytokine assay was replaced with zero in the statistical calculations. 

### 4.6. DPPH Scavenging Activity

The DPPH free radical scavenging activity of the natural plant extracts was estimated according to the method described by Hanato et al. (1988) [37] with modifications. AF-343 and the extracts of *Ulmus pumila* L., *Cassia tora* L., and *Taraxacum officinale* were diluted in distilled water at different concentrations, and then 180 μL of each dilution was added to a 0.2 mM DPPH ethanolic solution. Each mixture was shaken well and incubated at room temperature for 30 min, and then the absorbance was measured at 517 nm. The antiradical activity (three replicates per treatment) is expressed as the IC_50_ (μg/mL), the dose required to cause 50% inhibition of radical activity. The activity of ascorbic acid was measured as a control. DPPH radical scavenging activity was calculated using the following formula:

DPPH radical scavenging activity (%) = [(A0 - A1) x 100]/A0(1)

where A0 is the background absorbance of the sample, and A1 is the absorbance of the sample. 

### 4.7. Measurement of Intracellular ROS Generation 

To detect intracellular ROS, RBL-2H3 cells were seeded onto a 96-well black culture plate at a density of 1 × 10^5^ per well. After overnight incubation, the cells were preincubated with various concentrations of extracts for 1 h and then treated with compound 48/80 (500 μg/mL) to induce oxidative stress for an additional 30 min. Finally, 25 μM DCFH-DA was added to each well, and then the fluorescence intensity was measured in bottom-read mode using a SpectraMax M3 system (Molecular Devices) at excitation and emission wavelengths of 485 and 535 nm, respectively.

### 4.8. Sample Preparation for Chromatography

The dried plants (*Ulmus pumila* L., *Cassia tora* L., and *Taraxacum officinale)* were boiled with distilled water (1:10; w/v) at 90 °C for 9 h. Each extract was filtered and lyophilized. Two hundred micrograms of the lyophilized extract was added to 2 mL of 50% methanol (chicoric acid), 100% methanol (aurantio-obtusin), or 5% acetonitrile (catechin), and then sonicated for 1 h to isolate chicoric acid, aurantio-obtusin, and catechin. Each solution was filtered through a 0.45 µm membrane filter prior to injection into HPLC–UV system.

### 4.9. HPLC–UV Analysis

Catechin, aurantio-obtusin, and chicoric acid were identified and quantified in the extracts of *Ulmus pumila* L., *Cassia tora* L., and *Taraxacum officinale*, and in AF-343 using HPLC–UV (Agilent 1200 series, Agilent, USA; or SHIMADZU Prominence, Shimazu, Japan). Chromatographic separation was performed using various gradients or isocratic elution systems under different mobile phases and wavelengths to establish the optimal separation conditions for each chemical (Figures S1–S3). The column used was a Capcell-Pak UG120 C18 column (5 µm, 4.6 × 250 mm, Shiseido, Japan) with a flow rate of 1.0 mL/min. The sample injection volume was 20 µL.

### 4.10. Statistical Analysis

The values are shown as the mean ± SD. Analyses were performed using SPSS ver15.0.0 (SPSS Inc., Chicago, IL, USA). The statistical significance of differences among multiple groups was determined by one-factor analysis of variance (ANOVA) followed by Tukey’s or Dunnett’s T3 post hoc test. Values of p < 0.05 were considered to indicate statistical significance.

## Figures and Tables

**Table 1:** Content of catechin, aurantio-obtusin, and chicoric acid in AF-343 and extracts of *Ulmus pumila* L., *Cassia tora* L., and *Taraxacum officinale*.

	Catechin, g/kg (mean ± SD)	Aurantio-Obtusin, g/kg (mean ± SD)	Chicoric Acid, g/kg (mean ± SD)
AF-343	0.40 ± 0.01	1.60 ± 0.09	0.76 ± 0.01
*Ulmus pumila* L.	0.32 ± 0.02	ND	ND
*Cassia tora* L.	ND	0.49 ± 0.03	ND
*Taraxacum officinale*	ND	ND	11.19 ± 0.01

ND, not determined.

## Supplementary Materials

The following are available online, HPLC data for catechin, aurantio-obtusin, and chicoric acid identified in the three individual extracts of AF-343 in Figures S1–S3.
