# Supplementary material for: Inhibitory Effects of AF-343, a Mixture of Cassia tora L., Ulmus pumila L., and Taraxacum officinale, on Compound 48/80-Mediated Allergic Responses in RBL-2H3 Cells
Source: Molecules. 2020 May 22;25(10):2434. doi: 10.3390/molecules25102434 (PMC7288106; doi:10.3390/molecules25102434)
Supplement: Supplementary file 1 [file molecules-25-02434-s001.pdf]

Supplementary information

Method

HPLC condition

a) Catechin

The mobile phase was water : acetonitrile (99:5; v/v) with 0.1% trifluoroacetic acid (A) and water : acetonitrile (50:50; v/v) with 0.1% trifluoroacetic acid (B) with gradient elution procedures as follows: 10%-20% B, 0–10 min; 10% B, 10–20 min; 20%–50% B, 20-35 min; 50% B, 35-42 min; 50-10%B, 42-45 min; 10%B, 45-60 min. The wavelength for UV detection was set at 280 nm.

b) Aurantio-obtusin

The mobile phase was water: formic acid (99:1; v/v) (A) and pure methanol (B) containing 60% B for 30 min. The wavelength for UV detection was set at 286 nm.

c) Chicoric acid

The mobile phase was water: formic acid (99:1; v/v) (A) and pure methanol (B) with gradient elution procedures as follows: 20%-30% B, 0–15 min; 30%–100% B, 15–16 min; 100%–20% B, 16-26 min; 20% B, 26-30 min. The wavelength for UV detection was set at 320 nm.

A) Catechin

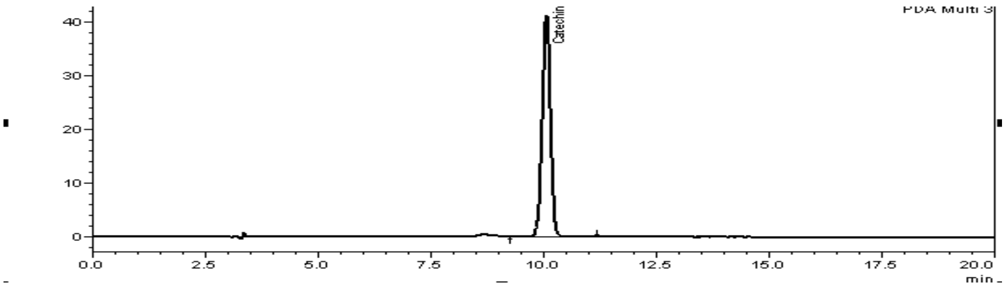

B) AF-343

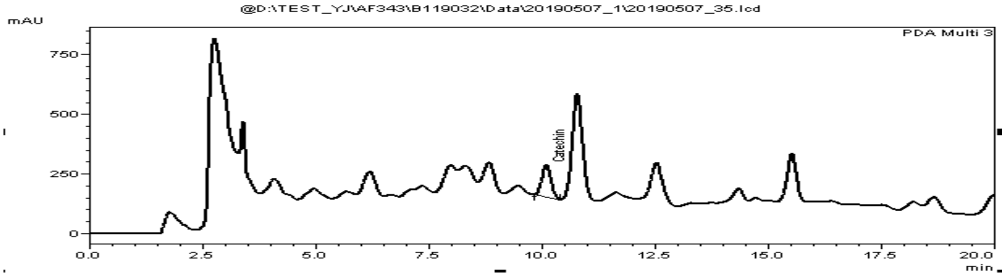

Figure S1. Chromatograms of (A) catechin standard solution and (B) catechin isolated from AF-343

A) Aurantio-obtusin

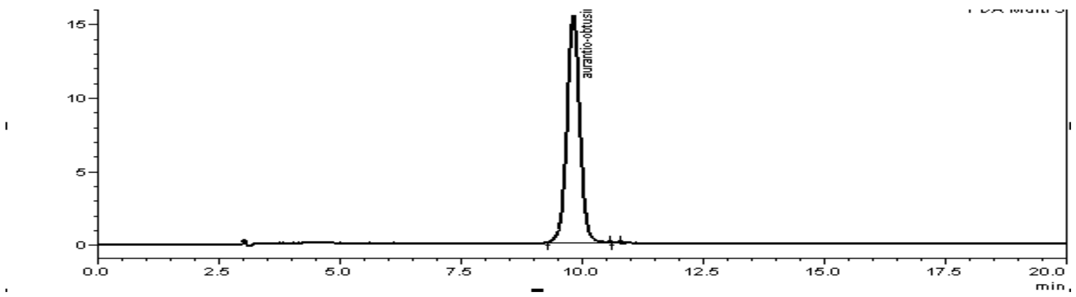

B) AF-343

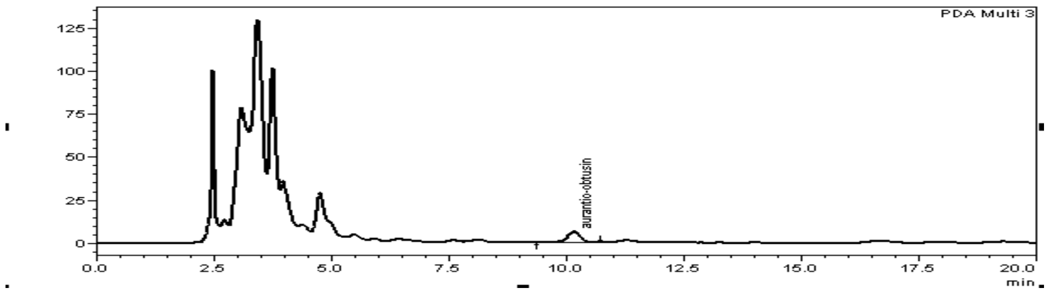

Figure S2. Chromatograms of A) aurantio-obtusin standard solution and B) aurantio-obtusin isolated from AF-343

A) Chicoric acid

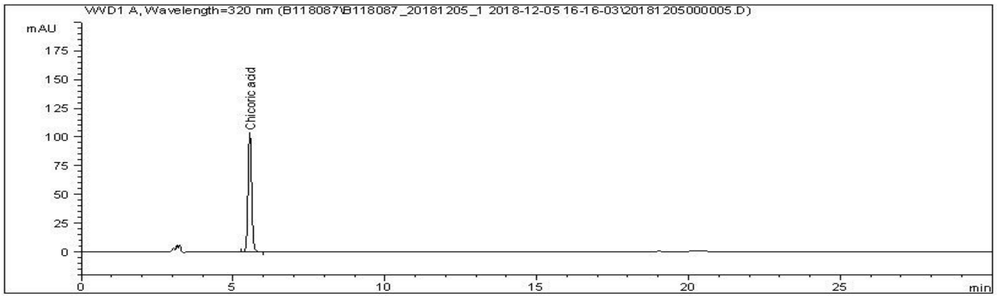

B) AF-343

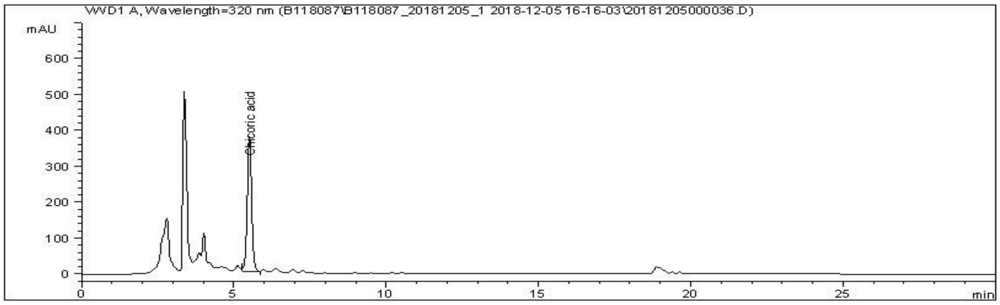

Figure S3. Chromatograms of A) chicoric acid standard solution and B) chicoric acid isolated from AF-343
